# Somatostatin-Expressing Inhibitory Interneurons in Cortical Circuits

**DOI:** 10.3389/fncir.2016.00076

**Published:** 2016-09-29

**Authors:** Iryna Yavorska, Michael Wehr

**Affiliations:** Institute of Neuroscience and Department of Psychology, University of OregonEugene, OR, USA

**Keywords:** somatostatin, Martinotti, GIN, x94, x98, VIP, parvalbumin, disinhibitory

## Abstract

Cortical inhibitory neurons exhibit remarkable diversity in their morphology, connectivity, and synaptic properties. Here, we review the function of somatostatin-expressing (SOM) inhibitory interneurons, focusing largely on sensory cortex. SOM neurons also comprise a number of subpopulations that can be distinguished by their morphology, input and output connectivity, laminar location, firing properties, and expression of molecular markers. Several of these classes of SOM neurons show unique dynamics and characteristics, such as facilitating synapses, specific axonal projections, intralaminar input, and top-down modulation, which suggest possible computational roles. SOM cells can be differentially modulated by behavioral state depending on their class, sensory system, and behavioral paradigm. The functional effects of such modulation have been studied with optogenetic manipulation of SOM cells, which produces effects on learning and memory, task performance, and the integration of cortical activity. Different classes of SOM cells participate in distinct disinhibitory circuits with different inhibitory partners and in different cortical layers. Through these disinhibitory circuits, SOM cells help encode the behavioral relevance of sensory stimuli by regulating the activity of cortical neurons based on subcortical and intracortical modulatory input. Associative learning leads to long-term changes in the strength of connectivity of SOM cells with other neurons, often influencing the strength of inhibitory input they receive. Thus despite their heterogeneity and variability across cortical areas, current evidence shows that SOM neurons perform unique neural computations, forming not only distinct molecular but also functional subclasses of cortical inhibitory interneurons.

## Introduction

Inhibitory interneurons represent about 20–30% of all cortical cells in mammals ranging from mice to humans (Hendry et al., 1987; Tamamaki et al., 2003; Markram et al., 2004; Sherwood et al., 2010). Interneurons exhibit remarkable diversity in their morphology, histochemistry, intrinsic membrane properties, and connectivity. This diversity strongly suggests that different types of interneurons play distinct roles in cortical computation, although only the first glimmers of these functional roles have so far been brought to light. Although inhibitory interneurons can be classified by many different characteristics, a widely used approach is to identify unique molecular markers such as neuropeptides or calcium binding proteins. This method has gained increasing popularity in recent years, because the promoters for such cell-type-specific genes provide access for targeting the expression of genetic tools to specific subsets of cells. Based on histochemical markers, we can divide cortical inhibitory cells into three non-overlapping categories in mice: those that express parvalbumin (PV), somatostatin (SOM), or vasointestinal peptide (VIP). These categories vary across species; in rats, for example, PV, SOM, and calretinin (CR) cells form non-overlapping categories (Gonchar and Burkhalter, 1997; Kawaguchi and Kubota, 1997), whereas mice show overlapping expression of SOM and CR (Freund and Buzsáki, 1996; Somogyi and Klausberger, 2005; Ascoli et al., 2008; Fishell and Rudy, 2011; Rudy et al., 2011). While these 3 major classes don't account for all inhibitory interneurons (a small number of interneurons express other less common markers), these 3 major classes do account for the vast majority (80–90%) of all inhibitory cells (Gonchar and Burkhalter, 1997; Rudy et al., 2011; Pfeffer et al., 2013).

In this review, we focus on SOM cells in cerebral cortex, with an emphasis on mice. For an excellent recent review of SOM interneurons in cortical circuits, see Urban-Ciecko and Barth (2016). Because SOM cells differ markedly in many respects from PV and VIP cells, we will briefly review some of the distinctive characteristics of those cell types. PV cells are by far the largest category of inhibitory cells, representing 30–50% of all inhibitory interneurons (Tamamaki et al., 2003; Rudy et al., 2011). Although PV cells are not a homogenous population, they do appear to share several features. PV cells are found throughout cortical layers 2–6 and are typically fast spiking (FS) cells with narrow spike waveforms. However, not all PV cells are FS cells, and not all FS cells are PV cells (Cauli et al., 2000; Markram et al., 2004; Moore and Wehr, 2013). PV cells tend to target the somata and proximal dendrites of both excitatory cells and other PV cells (Kubota et al., 2016). They provide powerful inhibition, but since they form depressing synapses, this inhibition is relatively short-lived (Beierlein et al., 2003). Although it is still unclear whether PV cells perform similar functions in different sensory regions, current evidence suggests that they most likely provide gain control in cortical networks by indiscriminately pooling locally available excitatory input and feeding this back to both PNs and other PV cells (Brumberg et al., 1996; Gabernet et al., 2005; Higley and Contreras, 2006; Cruikshank et al., 2010; Moore and Wehr, 2013). In sensory cortical areas with columnar organization, PV cells are thought to pool local input from similarly-tuned PNs, and are therefore well-tuned. For example, auditory cortex has columnar organization for sound frequency and PV cells there are well-tuned for frequency (Moore and Wehr, 2013). In contrast, mouse visual cortex does not have columnar organization for orientation, and PV cells pool input from heterogeneously-tuned PNs, and are therefore more broadly tuned for orientation than PNs (Niell and Stryker, 2008; Kerlin et al., 2010; Atallah et al., 2012).

**Figure 1:**
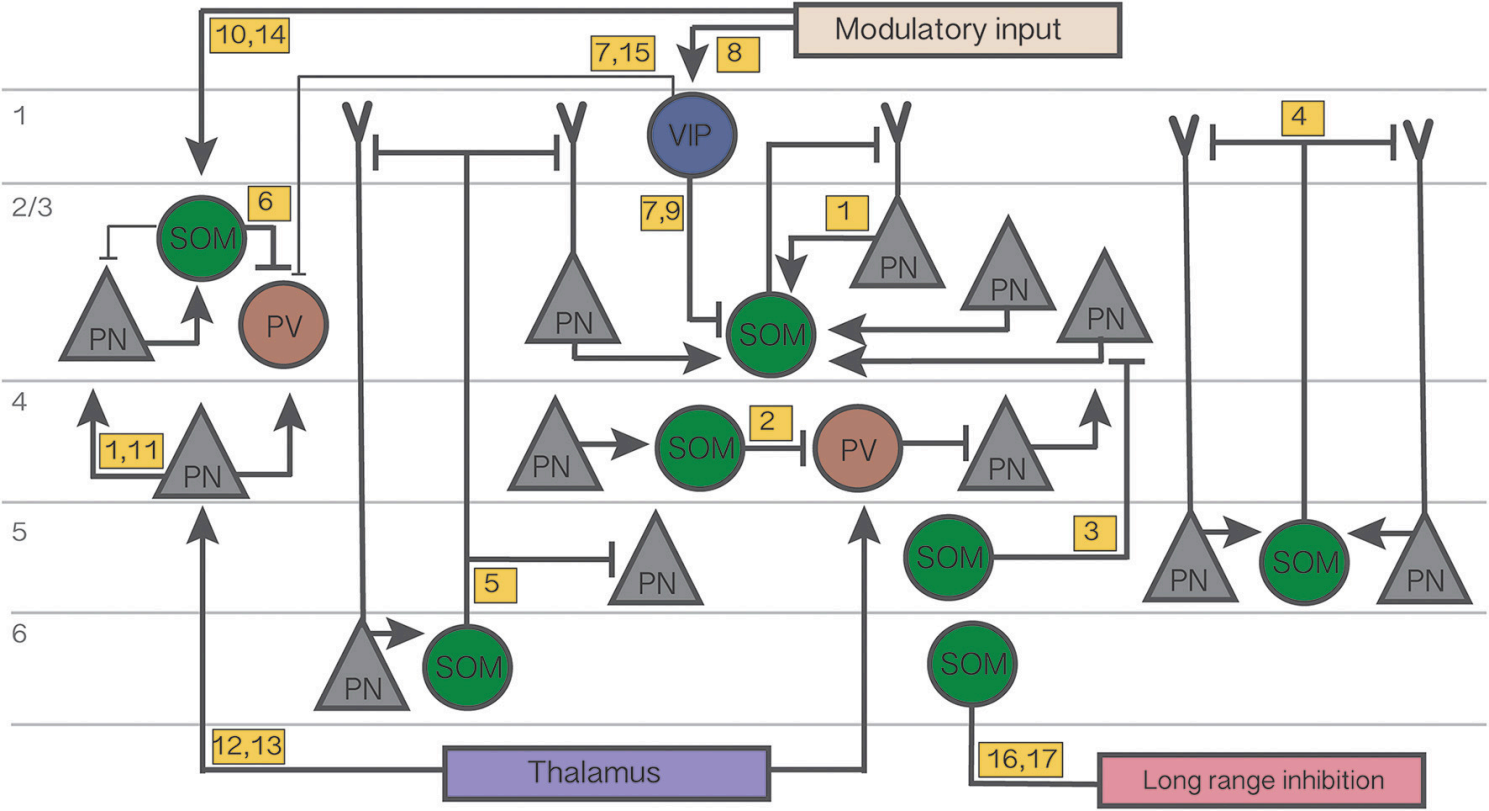
**Summary diagram of the cortical circuits in which SOM cells participate**. Numbers in yellow boxes refer to the references below. Some microcircuits combine results from different studies and include speculative connections. Connections not mentioned in the main text or not pertaining to SOM networks are omitted for simplicity. Thin lines indicate weaker connections. Figure references: 1. Adesnik et al. (2012); 2. Xu et al. (2013); 3. Kapfer et al. (2007); 4. Silberberg and Markram (2007); 5. Wang et al. (2004); 6. Cottam et al. (2013); 7. Pi et al. (2013); 8. Fu et al. (2014); 9. Karnani et al. (2016a); 10. Kawaguchi et al. (1997); 11. Li et al. (2015); 12. Cruikshank et al. (2010); 13. Beierlein et al. (2003); 14. Fanselow et al. (2008); 15. Letzkus et al. (2011); 16. Tomioka et al. (2005); 17. Endo et al. (2016).

While VIP cells comprise only 1–2% of all cortical cells, recent studies in a number of cortical areas have revealed that VIP cells provide weak inhibition to PV networks and strong inhibition to SOM networks, and thus indirectly regulate the activity of the local population of PNs (Lee et al., 2012; Hioki et al., 2013; Pfeffer et al., 2013; Pi et al., 2013; Karnani et al., 2016a). In the context of understanding SOM networks, VIP cells are of particular interest because they target SOM cells strongly in layers 2/3 (and also weakly in layer 5), forming robust disinhibitory circuits (Lee et al., 2013; Pfeffer et al., 2013; Karnani et al., 2016a; Figure 1). These disinhibitory circuits appear to be engaged under specific behavioral conditions including associative learning, reinforcement, locomotion, and attention (Uematsu et al., 2008; Letzkus et al., 2011; Pi et al., 2013; Fu et al., 2014; Kepecs and Fishell, 2014; Pala and Petersen, 2015). The axons of VIP cells extend vertically within a narrow column, thereby inhibiting mainly local SOM cells. VIP cells may therefore “open holes in the blanket of inhibition” that is provided by SOM cells to local PNs (Karnani et al., 2016a). VIP cells also belong to a subgroup of neurons that express the 5HT3a serotonin receptor, which also includes neurogliaform and late-spiking as well as a subset of cholecystokinin (CCK), CR, or neuropeptide Y (NPY) expressing neurons (Lee et al., 2010; Rudy et al., 2011).

## How many distinct kinds of SOM cells are there?

SOM cells compose 30% of all inhibitory cells in the cortex (Xu and Callaway, 2009; Rudy et al., 2011), and these can be further subdivided into smaller distinct groups based on layer, physiology, morphology, co-expression of other markers, and synaptic targets. These approaches typically produce partially overlapping categories, producing an inevitable tension between the tendency to be a “lumper” or a “splitter.” Anatomically, for example, the most distinctive type of SOM cell is the Martinotti cell (Martinotti, 1889; Karube et al., 2004; Wang et al., 2004; Ma et al., 2006). The most striking feature of Martinotti cells is their characteristic axonal projection to layer 1, where they make extensive lateral arborizations (Wang et al., 2004; Ma et al., 2006; Gentet, 2012). All Martinotti cells are SOM-positive, however not all SOM-positive cells are Martinotti cells. Martinotti cells are mostly located in supragranular layers 2 and 3, but can also be found sparsely in layers 4, 5, and 6. Their dendrites branch locally or down to deeper layers (Wang et al., 2004). Because Martinotti cells make up the largest and best-studied category of SOM cells, it is tempting to to lump together all other SOM cells as “non-Martinotti,” a category that would include multiple anatomical classes such as subsets of basket cells, bitufted, horizontal, and multipolar cells as well as long-projecting GABAergic neurons (Rogers, 1992; Reyes et al., 1998; Ma et al., 2006; McGarry et al., 2010; Suzuki and Bekkers, 2010; Kubota et al., 2011).

A complementary categorization approach has been to take advantage of transgenic mouse lines such as the GIN, X94, and X98 lines (Ma et al., 2006). These three different lines of transgenic GAD67-eGFP mice were generated by pronuclear injection (i.e., not by knock-in), and fortuitously label subsets of SOM cells (most likely due to insertional effects depending on where GAD67-eGFP randomly inserted into the genome). These lines are an excellent tool for restricting GFP expression to SOM cells. But because they label only subsets of SOM cells, one must be careful not to infer relative proportions of SOM subtypes from studies using these lines. The GIN line labels mostly Martinotti cells, and most of these are found in L2/3, with sparse labeling in L5. GIN cells account for 35% of SOM cells (Oliva et al., 2000; Ma et al., 2006). Targeted patching of these cells reveals that most of them have intrinsic firing properties characteristic of regular-spiking (RS) cells with generally depolarized membrane potentials, which distinguishes them from other types of SOM cells (Ma et al., 2006; McGarry et al., 2010; Kinnischtzke et al., 2012). L2/3 GIN cells are also likely to be electrically coupled to each other (with 66% likelihood) and are strongly activated by cholinergic signaling (Fanselow et al., 2008).

The X98 line labels Martinotti cells in L5 and upper L6, accounting for 20% of all SOM cells. X98 cells have distinctive intrinsic firing properties −40% of them are low-threshold spiking (LTS) cells. These cells are neither fast-spiking nor regular-spiking, but instead fire a characteristic rebound spike when depolarized from a relatively hyperpolarized holding potential, often in bursts. All LTS cells are inhibitory, but only about half of LTS cells are SOM-positive, and of those most are Martinotti cells (Gibson et al., 1999; Beierlein et al., 2003; Goldberg et al., 2004). Morphologically, Martinotti cells in L5 are mostly similar to Martinotti cells in L2/3, except for a tendency to send their axons either to L4 or deeper layers in addition to L1 (Wang et al., 2004).

The X94 line labels only non-Martinotti cells in layers 4 and 5a. Thus X94 cells are a completely distinct population from GIN and X98 cells, whereas the GIN and X98 populations partially overlap with each other. X94 cells account for about half of L4 SOM cells and have a basket-like morphology with mostly local axonal projections (unlike the striking L1 projection seen in Martinotti cells). Unlike Martinotti cells, which target PNs, X94 cells target PV-positive FS interneurons in layer 4. X94 cells fire narrow action potentials at high rates, and therefore resemble FS cells, but unlike FS cells they have a distinctive “stuttering” firing pattern in which their spike trains are interrupted by seemingly random periods of silence. These firing properties have therefore been called “FS-like” or “quasi-FS” (Ma et al., 2006; Large et al., 2016). In piriform cortex, SOM-positive cells can also exhibit tonic fast spiking firing properties and PV-positive neurons can be stuttering fast spiking, which indicates that different cells types can have similar firing properties and thus this criterion alone can not be used to reliably identify a specific cell type (Large et al., 2016). Interestingly, the SOM cells in layer 4 that are not labeled in the X94 line are similar to X94 cells in all of these respects: they have only local axons that target L4 PV-positive cells, and have the stuttering FS-like firing type. Thus it appears that L4 SOM cells may be a single population, with no apparent functional correlates of their segregation into X94 and non-X94 categories (Ma et al., 2006; McGarry et al., 2010; Xu et al., 2013). However, some Martinotti cells are found sparsely in L4, at least in rat (Wang et al., 2004).

Another genetic tool increasingly used to investigate SOM neurons is the SOM-Cre line (Taniguchi et al., 2011; Lovett-Barron et al., 2012), which allows researchers to use optogenetic or other Cre reporters to manipulate or record SOM cell activity across cortical layers. The SOM-Cre lines target all SOM cells, and have recently been used to study a number of specific brain regions (Cottam et al., 2013; Polack et al., 2013; Chen I.-W. et al., 2015; Neske et al., 2015; Sturgill and Isaacson, 2015). It is important to note that at least one SOM-Cre line (Taniguchi et al., 2011; Jax Stock No. 013044) also erroneously marks a small subset (6–10%) of fast spiking PV neurons (Hu et al., 2013), perhaps due to transient SOM expression during embryonic development in a small subpopulation of FS PV cells (although this has not yet been tested). It is not yet known whether this is also true for the other SOM-Cre line (Lovett-Barron et al., 2012). It is also important to note that about 5% of GFP-expressing neurons in the X94 and X98 lines, and 3% of cells in the GIN line, are not SOM-positive (Ma et al., 2006). This could be attributed to low expression of SOM in some cells which might be below the detectability threshold for immunohistochemistry, highlighting the point that immunohistochemistry results should be interpreted with caution. Although the percentages of this off-target labeling are low, they should still be taken into consideration in experiments. More generally, it is important to note that immunohistochemistry is a relatively difficult technique, and standards for antibody validation have been adopted relatively recently. This likely contributes to sometimes contradictory results for peptide expression in different studies.

A small subset (6–9%) of SOM cells that express NPY, nitric oxide synthase (NOS), and the Substance P receptor (SPR) form a distinct morphological class with long distance axonal projections. Although few in number, they can project to multiple brain regions both horizontally and vertically, making them good candidates for synchronizing neural activity across multiple cortical and subcortical regions (Tomioka et al., 2005; Kubota et al., 2011, 2016; Caputi et al., 2013; Kubota, 2014; Endo et al., 2016). These cells have high spine density early in development, which is greatly reduced during maturation (Kubota et al., 2011). Additionally, since NOS-positive neurons are highly active during sleep, while most SOM neurons are not, SOM/NOS/SPR neurons are likely to form a distinct subclass with different morphology and activity patterns (Kilduff et al., 2011; for review see Tricoire et al., 2012).

**Figure 2:**
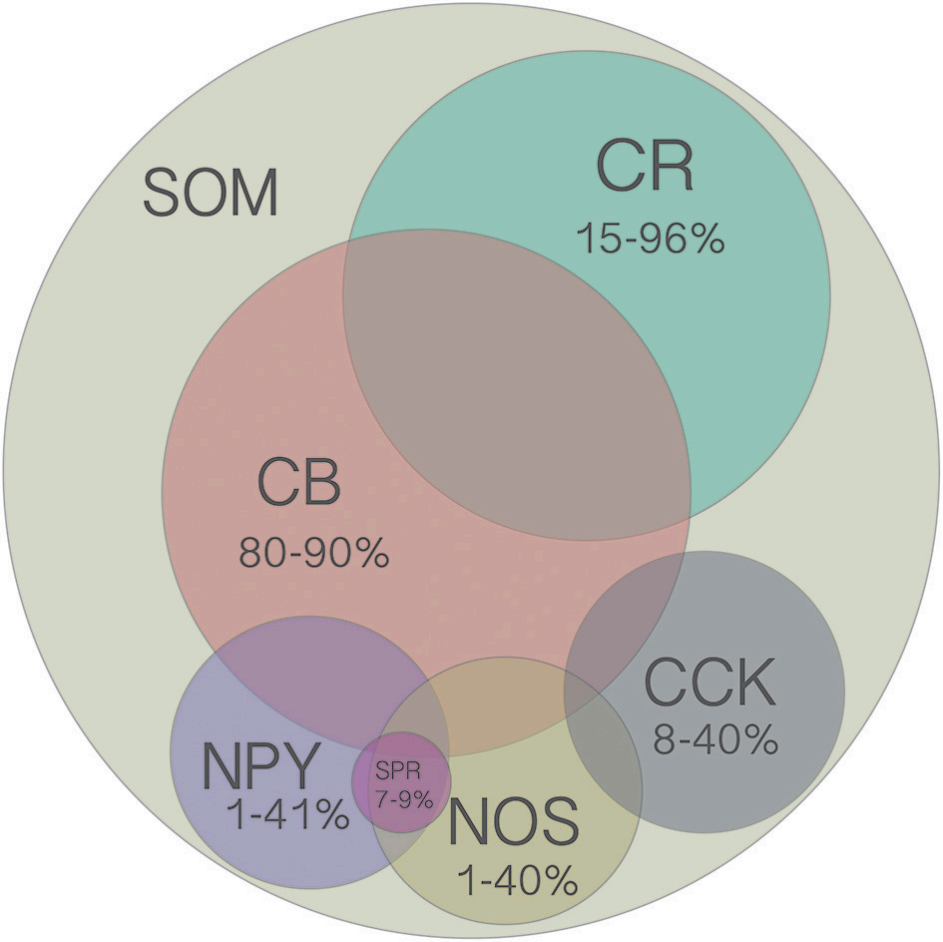
**Venn diagram of all molecular markers that are colocalized with SOM (CB, CR, NPY, NOS, CCK)**. Numbers indicate the range of reported percentages of SOM-positive cells that coexpress a given marker across studies in different species (mouse or rat), cortical layer (2–6), and cortical region (frontal, visual, or somatosensory). For more details, see Table 2. Area of each circle approximately represents the average range. Overlap of circles indicates known coexpression of depicted markers, however the area of the overlap does not indicate the extent of coexpression. Although there are no reports of SOM cells coexpressing more than two other molecular markers, this possibility has not been ruled out since it was rarely tested for.

Adding to this diversity are distinct laminar distributions of many of these cell types, as detailed below. In addition, SOM cells co-express a variety of other molecular markers such as CB (calbindin; Kubota and Kawaguchi, 1994; Wang et al., 2004; Ma et al., 2006; Suzuki and Bekkers, 2010), NPY (Kubota and Kawaguchi, 1994; Ma et al., 2006), CR (Xu et al., 2006; Xu and Callaway, 2009), CCK (Gonchar and Burkhalter, 1997), NOS (Kubota and Kawaguchi, 1994; Gonchar and Burkhalter, 1997; Kawaguchi and Kubota, 1997; Xu et al., 2006; Kilduff et al., 2011; Perrenoud et al., 2012), and SPR (Tomioka et al., 2005; Kubota et al., 2011, 2016; Caputi et al., 2013; Figure 2). Because most studies have used only a subset of these categorization methods (indeed, some methods are mutually exclusive, such as the transgenic lines), it is still not clear how many distinct combinatorial types of SOM cells are found in the cerebral cortex. Nevertheless, it may be informative to attempt to estimate upper and lower bounds on the number of distinct SOM subtypes. At a minimum there are 4 distinct types: (1) L2/3 Martinotti cells, which are mostly regular-spiking, labeled by GIN, and target PNs, (2) L5 Martinotti cells, which include LTS, are labeled by X98, and target PNs, (3) L4 SOM cells, which are FS-like and target L4 PV+ cells, and some of which are labeled by X94, and (4) NOS/SPR long-projecting GABAergic cells that can make either cortico-cortical or corticofugal projections (Table 1). This estimate undoubtedly lumps together subtypes of cells that can be distinguished by at least some criteria, but represents a lower bound on the number of functionally distinct SOM subtypes.

**Table 1:** **Four main subtypes of SOM cells (including 3 labeled by specific transgenic lines) with a minimal degree of overlap**.

**Line**	**Morphology**	**Layer**	**Firing properties**	**Marker expression**
GIN	Martinotti	L2/3, L5	RS	30% CB
				27% NPY
X94	Non-Martinotti	L4, L5	FS-like	None
X98	Martinotti	L5	LTS	96% CB
				40% NPY
None	Non-Martinotti	L2, L5+6	RS/BS	100% NPY+NOS+SPR
	Long-projecting		LTS	

*Subtype differences are based on morphology, cortical layer, firing properties, and co-expressed markers. Numbers in the Marker Expression column are the percentage of cells labeled in that subtype that express a given marker*.

What about the other extreme, an upper bound estimate of the maximum possible number of SOM cell types? Estimating such an upper bound requires several assumptions about whether classification criteria can vary independently (producing all possible combinations) or whether there are correlations that reduce the number of combinations. These assumptions are poorly constrained by data because most studies have measured only a subset of these criteria. In addition, we assume here that these criteria are static, although it's likely that some criteria could change over time. For example, cellular firing properties (such as bursting) can change under certain conditions in some cells (Bahrey et al., 2002), and molecular expression likely changes during development. Based on the ranges of classification criteria (detailed in Table 2), we estimated an upper bound of 100 on the number of possible subtypes of SOM cells. For example, in layer 2/3 there are two types of firing properties (RS and bursting) and 6 molecular markers (CB, CR, NPY, CCK, NOS, and SPR) in SOM cells. These belong mostly to one of two morphological classes, Martinotti cells, and multipolar long-projecting neurons that express NOS/SPR. Cells are exclusively RS or bursting, but molecular markers can be expressed in various combinations (but are rarely tested for co-expression, providing little constraint on the number of possible combinations that could occur in SOM cells). Thus there are 2 morphological types, 2^6^ potential combinations of molecular markers, and 2 firing-property classes, leading to 256 possible subtypes of SOM cells (2·2^6^·2). Of these 256 possible combinations, bursting cells are known not to express NPY, and there is evidence against co-expression for 6 of the 15 possible binary combinations of markers (Table 3). This puts an upper bound of 25 on the number of potential SOM subtypes in L2/3. Following similar logic for layers 2–6 (see Table 2 for details), we estimate the overall number of distinct subtypes of cortical SOM cells is likely no greater than 100. Additional data that further constrains the number of possible combinations would reduce this number. Thus while the true number of distinct subtypes of SOM cells is unknown, we estimate that it most likely lies somewhere between 4 and 100 subtypes.

**Table 2:** **Diversity of SOM cells across cortical layers 2–6**.

**Layer**	**Co-expression**	**Morphology**	**Firing properties**	**Targets**	**Max Number**
2/3	CB^6^ (32–92%^7^; 93%^9^; 20%^11^;86%^2^; 85%^17^)	Martinotti^2^, ^5^, ^7^, ^13^, ^16^, ^18^, ^19^, ^21^, ^22^	RS^5^, ^7^	L2/3 PNs^1^, ^18^	25
	NPY^6^ (15–27%^7^; 18%^11^)	Non-Martinotti^20^, ^21^, ^22^, ^23^	Bursting^11^	L2/3 INs^1^	
	CR (57–96%^12^, ^13^, ^22^)				
	CCK (10%^2^, ^11^, ^22^)				
	NOS^8^, ^20^, ^24^ (0.5%^2^; 1–40%^12^)				
	SPR^23^, ^20^ (6.6%)				
4	CB (31%^11^)	Non-Martinotti^7^, ^18^, ^19^	LTS^14^, ^15^	L4 INs^18^	9
	NPY (5%^11^)	Martinotti^2^, ^11^	FS-like^7^	L4 PNs^14^	
	CR (10%^11^; 18–44%^12^, ^22^)		Bursting^11^		
	CCK (8%^2^, ^11^, ^22^)				
5	CB^5^, ^6^ (49–96%^7^;15%^11^;92%^9^;86%^2^;92%^17^)	Martinotti^2^, ^10^, ^16^, ^18^, ^19^, ^21^, ^22^, ^23^	RS(<50%)^5^, ^7^, ^22^	L5 PNs^10^	27
	NPY(1.4–41%^7^; 40%^11^)	Non-Martinotti^7^, ^21^	LTS (<50%)^3^, ^7^	L2/3 PNs^4^	
	CR(12–19%^12^, ^22^)		FS-like^7^		
	NOS^8^, ^20^, ^24^ (0.5%^2^; 1–40%^12^)		Bursting^5^, ^11^, ^22^		
	SPR^23^ (8.6%)				
6	CB^6^ (49–96%^7^;92%^9^;86%^2^;92%^17^)	Non-Martinotti^9^, ^21^, ^23^	RS^11^	PNs^15^	39
	NPY^6^ (1.4–41%^7^; 40%^11^)	Martinotti^2^, ^7^, ^11^, ^19^, ^21^	LTS^15^		
	CR (15–17%^12^, ^22^)		Bursting^11^		
	CCK (40%^2^, ^11^, ^22^)				
	NOS^8^, ^20^.^24^ (0.5%^2^; 1–40%^12^)				
	SPR^23^, ^20^ (8.4%)				
Total					100

*No SOM cells are found in L1. The Co-expression column lists known molecular markers that co-localize with SOM in each layer, with the percentage of SOM cells that co-express each marker in parentheses. We included both bursting and LTS as distinct firing types, as reported in separate studies^3, 7, 11, 14, 15^, but considered these as a single category for the purposes of calculating an upper bound on the number of distinct SOM cell types (Max Number column). We determined the upper bound by counting the number of possible combinations of markers, morphology, and firing type, given the constraints on marker co-expression detailed in Table 3, known correspondences between morphology and firing properties, and the observations that bursting SOM cells don't express NPY and that L4 non-Martinotti (X94) cells don't express CB or NPY*.

1. Cottam et al. (2013) mouse, visual;

2. Gonchar and Burkhalter (1997) rat, visual cortex;

3. Goldberg et al. (2004) mouse, visual and somatosensory cortex;

4. Kapfer et al. (2007) mouse, somatosensory cortex;

5. Kawaguchi and Kubota (1996) rat, frontal cortex;

6. Kawaguchi and Kubota (1997) rat, frontal cortex;

7. Ma et al. (2006) mouse (GIN, X94, X98), somatosensory cortex;

8. Perrenoud et al. (2012) mouse, barrel cortex;

9. Rogers (1992) rat, visual cortex;

10. Silberberg and Markram (2007) rat, somatosensory cortex;

11. Wang et al. (2004) rat, somatosensory cortex;

12. Xu et al. (2006) mouse GIN, frontal (high % NOS), somatosensory, and visual cortex;

13. Xu and Callaway (2009) mouse GIN, somatosensory;

14. Beierlein et al. (2003) rat, barrel cortex;

15. Gibson et al. (1999) rat, somatosensory cortex;

16. Karube et al. (2004) rat, frontal cortex;

17. Kubota and Kawaguchi (1994) rat, frontal cortex;

18. Xu et al. (2013) mouse, somatosensory cortex;

19, McGarry et al. (2010) mouse, frontal, somatosensory, and visual cortex;

20. Endo et al. (2016) mouse, visual cortex;

21. Kawaguchi and Kubota (1998) rat, frontal cortex;

22. Uematsu et al. (2008) rat, frontal cortex;

23. Kubota et al. (2011) rat, frontal cortex;

24. *Tomioka et al. (2005) mouse, motor, somatosensory, and visual cortex*.

**Table 3:** **Co-expression of molecular markers in SOM cells**.

	**NPY**	**CR**	**NOS**	**CCK**	**SPR**
CB	+^7, 24^	+^12, 13^	+^2, 8, 24^	+^11^	+^23^
NPY		−^2^	+^8, 24^	−^11^	+^23^
CR			−^2^	−^2^	−^23^
NOS				+^8^	+^20, 23^
CCK					−^23^
SPR					

*Reported co-expression of binary combinations is indicated by +, reported absence of co-expression is indicated by −. Markers have been reported to co-express in at least some SOM cells for all binary combinations except NPY-CR, NPY-CCK, CR-NOS, CR-CCK, CR-SPR, and CCK-SPR. We pooled data from all layers and did not distinguish between NOS-1 and NOS-2. Numbers refer to references in Table 2*.

Another important means of categorizing SOM cells is by the layer in which their cell bodies reside. Here, we consider each of these layers in turn.

### Layer 2/3

Multiple studies have shown that layer 2/3 SOM cells provide strong inhibition to L2/3 PNs (Fino and Yuste, 2011; Adesnik et al., 2012; Cottam et al., 2013; Xu et al., 2013). However, there is disagreement over how much inhibition L2/3 SOM cells provide to other inhibitory cells in L2/3. In somatosensory cortex, L2/3 SOM neurons provide only weak inhibition to other inhibitory cells (Xu et al., 2013). A quite different pattern was observed in the visual cortex. There, SOM neurons strongly inhibit PV cells (twice as potently as PNs), and this inhibition causes PV cells to be more tuned to orientation (Cottam et al., 2013; Pfeffer et al., 2013). The difference between these findings may indicate different circuits in somatosensory and visual cortex, but it is important to note that these studies also used different methods. Xu et al. performed slice experiments using current injections in single SOM neurons and optogenetic suppression of SOM neurons, whereas Cottam et al. looked at neural firing rate *in vivo* in behaving animals with and without presentation of visual stimuli while optogenetically activating many SOM neurons. Considering that brain states can strongly affect neural activity, it is quite possible that the reported differences in the inhibitory contribution of L2/3 SOM cells to neighboring neurons could be explained by different cortical states and the number of SOM cells recruited.

L2/3 SOM cells avoid inhibiting each other, and instead receive most of their inhibition from VIP and PV cells (Pfeffer et al., 2013). Layer 2/3 SOM neurons have also been shown to participate in a form of lateral inhibition in visual cortex, pooling excitatory input from adjacent PNs and thereby contributing to surround suppression (Adesnik et al., 2012). Indeed, stimulation of a single L2/3 PN in visual cortex can activate 30% of SOM neurons within a 100 micron radius (Kwan and Dan, 2013). This activation of SOM cells by PNs was more strongly distance-dependent than PN → PN activation (Kwan and Dan, 2013). Activity of the SOM network also increases supralinearly as the number of active L2/3 PNs increases. Compared to activating a single PN, activation of just two L2/3 PNs causes a 10-fold increase in the strength of recruited SOM inhibition (Kapfer et al., 2007). Interestingly, in barrel cortex SOM cells are suppressed during stimulus presentation, and thus are anti-correlated with network activity, a feature which has not been observed in other sensory regions (Gentet et al., 2012). In contrast, strong activation of PNs is accompanied by a linear recruitment of PV-mediated inhibition (Xue et al., 2014). Thus, it appears that the most important contribution of SOM inhibition in L2/3 is likely to be driven by high-frequency activation of just a few surrounding PNs.

In visual and auditory cortex, L2/3 SOM cells respond much later than other neurons to input from L4, and have lower spontaneous firing rates than other inhibitory cells (Ma et al., 2010; Li et al., 2015). These late responses have been attributed to the fact that SOM cells in L2/3 do not receive input directly from L4, but rather pool from L2/3 PNs. In general, SOM cells respond with a delay, even if they are receiving input from neighboring L2/3 PNs (Kapfer et al., 2007; Kwan and Dan, 2013). This delay probably also arises in part from the integration of inputs from facilitating synapses.

L2/3 SOM neurons participate in both feedforward as well as feedback inhibition, and primarily target the dendrites of L2/3 pyramidal neurons (Karnani et al., 2016a). Paired *in vitro* recordings of SOM cells (in the GIN line) revealed that both their subthreshold and suprathreshold activity is highly synchronous and they exhibit persistent firing more frequently than other cell types (Fanselow et al., 2008). During spontaneous activity *in vivo*, however, SOM cells do not correlate with oscillatory network activity, whereas PV cells do (Kwan and Dan, 2013). These results support the idea that PNs and PV cells receive similar input (dominated by thalamic activation), whereas SOM cells are modulated by distinct pathways (including top-down and subcortical input).

### Layer 4

Most SOM cells in layer 4 are strikingly different from the typical Martinotti SOM cells in other layers. The X94 line sparsely labels L4 and L5a SOM neurons (Ma et al., 2006; Xu et al., 2013), and these neurons do not send their axons to layer 1, but instead target other inhibitory cells (i.e., PV cells) in layer 4. In fact, unitary IPSPs from SOM cells onto L4 PV cells are much larger than those in L4 excitatory cells (Xu et al., 2013). The morphology of L4 SOM cells also differs from other SOM neurons. They are typically described as bitufted or multipolar cells that keep their axons and dendrites in the same layer (Gonchar and Burkhalter, 1997; Ma et al., 2006). These cells rarely co-express other markers and have been characterized as either quasi-FS (Ma et al., 2006) or bursting (Kubota and Kawaguchi, 1994; Wang et al., 2004). While thalamocortical axons in L4 provide strong and direct input to pyramidal and fast spiking cells, L4 SOM cells are only weakly excited by thalamic input (Cruikshank et al., 2010). Synaptically coupled SOM-FS pairs in L4 of barrel cortex can show synchronous spiking activity, even in the presence of glutamate antagonists (but not when GABA_A_ receptors are blocked), indicating that inhibitory GABA_A_-mediated synaptic transmission is both necessary and sufficient to induce synchronous activity between SOM and FS cells (Hu et al., 2011). A small number of SOM cells with Martinotti morphology are also found in L4, at least in juvenile rat (Wang et al., 2004). Due to differences in their targets, it appears that FS-like and RS SOM cells are members of different circuits in L4. In the frontal cortex, these cells also show different patterns of activity during a foraging task (Kvitsiani et al., 2013).

### Layer 5

Layer 5 SOM cells represent 19% of all inhibitory cells in L5, some of which also co-express NPY and/or CB (Kawaguchi and Kubota, 1997; Ma et al., 2006) and which are labeled in the X98 mouse line. Layer 5 pyramidal neurons form disynaptic inhibitory circuits with one another via the L5 SOM network (Silberberg and Markram, 2007). Activation of a L5 PN typically produces inhibition in neighboring PNs, and 40–90% of this inhibition comes from a single L5 Martinotti cell (Silberberg and Markram, 2007). Layer 5 SOM cells also inhibit a subpopulation of L2/3 PNs, consistent with the translaminar projections that are the hallmark of Martinotti cells (Kapfer et al., 2007; Xu and Callaway, 2009). 50% of SOM cells in L5 are low-threshold spiking cells. LTS SOM cells differ in their connectivity to one another compared with other SOM neurons. About 40% of LTS SOM cells make inhibitory connections with one another (Fino and Yuste, 2011). A small percentage of X94 line SOM neurons are also found in L5a (Ma et al., 2006). These neurons create a disinhibitory network by targeting PV cells and, at least in motor cortex, hyperactivity of SOM cells in L5 leads to excitotoxicity and death of excitatory neurons (Zhang et al., 2016).

### Layer 6

SOM neurons in layer 6 consist mainly of Martinotti cells that coexpress variable combinations of molecular markers such CB and NOS in rat (Kubota and Kawaguchi, 1994) as well as NPY and CCK in mouse (Wang et al., 2004). These cells send axons to layer 1, but about half of the SOM cells in L6 also make axonal arborizations in layers 5 and 6, suggesting less specific laminar targeting than layer 2/3 Martinotti cells (Wang et al., 2004; Ma et al., 2006).

Several studies have now identified a small population of GABAergic projection neurons, i.e., GABAergic inhibitory neurons that are not interneurons (McDonald and Burkhalter, 1993; Gonchar et al., 1995; Tomioka et al., 2005). These cells, while few in number (only 7–9% of SOM cells), project axons outside of the local area, can travel up to several mm, can cross areal boundaries, and in some cases project through the corpus callosum to the contralateral hemisphere (Gonchar et al., 1995). The vast majority of these cells express SOM, NPY, NOS, and substance P receptor (SPR), and are found in layer 6 (and to a lesser extent, in L2 and L5; Tomioka et al., 2005; Kubota et al., 2011; Caputi et al., 2013). Recent work has shown that these long inhibitory projections can regulate the output of medium spiny neurons in striatum, thereby modulating the activity of both direct (D1-type dopamine receptors) and indirect (D2-type dopamine receptors) reward pathways (Rock et al., 2016). NOS-positive projection neurons are a small subpopulation of neurons that is active during slow wave sleep, suggesting that they could play a major role in homeostatic sleep regulation by influencing global neuronal activity (Kilduff et al., 2011; for review see Tamamaki and Tomioka, 2010).

### Firing properties

Cortical SOM cells differ in their firing properties and electrophysiology. In particular, several distinct categories have been reported, including regular spiking (RS) cells, LTS, bursting, and FS-like or stuttering cells (Kawaguchi and Kubota, 1996; Goldberg et al., 2004; Wang et al., 2004; Ma et al., 2006; Fanselow et al., 2008; Large et al., 2016). There is some disagreement about the prevalence of FS-like or stuttering SOM cells in different layers; one possible reason for this is that FS-like SOM cells might have been categorized as FS cells in some studies (Beierlein et al., 2003; Wang et al., 2004; Ma et al., 2006). SOM cells have also been classified as either accommodating or non-accommodating (Wang et al., 2004). Accommodating cells (the vast majority, at 90% of SOM cells) include both RS and bursting types, whereas non-accommodating cells (only 8% of SOM cells) have been described as analogous to FS cells based on their ability to fire at high rates without adaptation. Regular spiking and bursting firing types were originally described for cortical pyramidal neurons (Connors et al., 1982), and it is important to note that the firing properties of RS or bursting SOM cells are analogous but not identical to those classically observed in pyramidal neurons. For example, *in vivo* whole cell studies show that average action potential waveform of SOM neurons is somewhat narrower than pyramidal cells and wider than fast spiking cells recorded both in CRE-IRES-SOM and GIN transgenic lines, thus putting them in a different category than RS neurons (Gentet et al., 2012; Polack et al., 2013). But in slice recordings from younger animals, the waveform of SOM neurons is comparable in width to excitatory neurons (McGarry et al., 2010). This discrepancy so far has not be investigated and could arise from either differences in recording methods or animal age.

It is still not clear whether LTS and bursting SOM cells form two distinct categories or instead are a single class that lie along a continuum. LTS cells are Martinotti cells, and are found in cortical layers 4, 5, and 6 (Kawaguchi and Kubota, 1996; Beierlein et al., 2000, 2003; Goldberg et al., 2004; Wang et al., 2004; Ma et al., 2006). They are so-named because they have very low thresholds for action potential initiation. Because of this low threshold, single-axon inputs to layer 5 LTS cells can generate spikes (Kozloski et al., 2001). In addition, LTS cells have a strong tendency to fire spikes or bursts on rebound from hyperpolarization. These rebound spikes/bursts are mediated by T-type calcium channels, similar to those found in thalamic relay neurons (Goldberg et al., 2004; Ma et al., 2006). Like relay neurons, L5 LTS cells can fire in either tonic or bursting mode, depending on their membrane potential. In contrast, L4 LTS cells fire tonically under control conditions, and only fire bursts in the presence of metabotropic glutamate agonists (Beierlein et al., 2000, 2003; Goldberg et al., 2004). This suggests that LTS cells in layer 4 and 5 probably form two distinct classes.

Upon release from hyperpolarization, LTS cells can fire either a single spike or a burst of spikes, which in some studies has led to them being categorized as two distinct groups (Wang et al., 2004; Ma et al., 2006). However, it is possible that this difference is due only to variation in input resistance along a continuum (Ma et al., 2006), which would instead suggests that they form only a single group. Different studies have adopted different terminology (either bursting or LTS), and report somewhat different laminar distributions, which is further complicated by the fact that some studies are in rat while others are in mouse (Wang et al., 2004; Ma et al., 2006). Bursting SOM cells exhibit a prominent after-depolarization, which is almost certainly mediated by an I_h_ current because these cells express HCN channel genes. Pharmacological blockade of the the I_h_ current in GIN and X94 cells eliminates rebound depolarization, indicating that I_h_ likely contributes to bursting in those types of SOM cells. But blockade of the low-threshold T-type calcium channel (but not I_h_) eliminates rebound bursting in only X98 cells, indicating that T-type channels are essential for this distinctive firing property of L5 LTS cells (Ma et al., 2006). Thus, based on the channels involved, LTS and bursting SOM cells described in different studies probably represent at least partially distinct populations. It seems likely that classification of SOM cells based on firing properties alone (such as the tendency to burst) could lump together distinct classes or erroneously split a single class, depending on the sample being studied.

LTS cells are notable because they have been shown to form gap-junction coupled networks in layer 4 of barrel cortex (Gibson et al., 1999; Beierlein et al., 2000; 2003 Amitai et al., 2002; Ma et al., 2011). Electrical coupling was also observed in L2/3 SOM neurons in the GIN line, although these are usually not categorized as LTS cells (Fanselow et al., 2008). Electrical coupling of SOM cells likely has a substantial impact on the net effect of the SOM network, because SOM inhibition increases supralinearly when more than one SOM neuron is activated (Kapfer et al., 2007). Adding to the importance of electrical coupling to SOM network activity is the fact that SOM cells don't receive thalamic input, and instead are mostly driven by intracortical input (Beierlein et al., 2003; Cruikshank et al., 2010). Although individual SOM neurons in L2/3 and L4 don't provide as strong or reliable inhibition to PNs as do PV cells (Beierlein et al., 2003; Pfeffer et al., 2013), together as a unified network they may act as a powerful inhibitory force when activity in the local cortical excitatory population increases.

### Synaptic physiology and input

Unlike strongly depressing PN → FS and FS → PN synapses, L4 and L2/3 SOM cells typically receive strongly facilitating synaptic input from PNs and weakly facilitating synaptic input from FS and VIP cells (Thomson, 1997; Thomson and Deuchars, 1997; Markram et al., 1998; Reyes et al., 1998; Ma et al., 2012; Pi et al., 2013; Karnani et al., 2016b). This suggests that SOM neurons are strongly but transiently inhibited at the onset of a new stimulus, but likely recover during prolonged activity. Facilitating input to SOM cells also means that they would be more sensitive to a sustained train of input from a single cell than to simultaneous but transient input from multiple PNs. Consistent with this, activation of SOM cells is quite different from other inhibitory cells depending on cortical network state. Synaptic input received by LTS SOM cells tends to be weaker and less reliable at low stimulation frequencies (<20 Hz) compared to input received by FS cells (Beierlein et al., 2003; Ma et al., 2012). At higher stimulation frequencies (>10–20 Hz), LTS cells are powerfully recruited at the same time that synapses onto other inhibitory cells become depressed. As a result, SOM cells are unlikely to be recruited during periods of low cortical activity, but become strongly activated during high cortical network activity. Congruent with these effects of network dynamics on SOM activation, SOM cells make weakly facilitating synapses onto both PNs and FS cells. Thus SOM-mediated inhibition tends to be weak and unreliable at low frequencies, but will become robust at higher frequencies, making SOM cells an important player in shaping neural responses to prolonged stimuli (Gupta et al., 2000; Beierlein et al., 2003; Kapfer et al., 2007; Hayut et al., 2011; Ma et al., 2012; Pfeffer et al., 2013). Tonic firing of SOM neurons regulates spontaneous activity of principal cells via slow GABA_B_ receptors (Urban-Ciecko et al., 2015), while also contributing fast GABA_A_-mediated synaptic input onto the distal dendrites of PNs (Wang et al., 2004; Silberberg and Markram, 2007). This indicates that SOM neurons contribute to spontaneous and evoked cortical responses.

Input to SOM cells is also unique since it does not appear to follow the canonical pattern of ascending thalamocortical information flow. Activation of thalamic fibers evokes a strong feedforward and depressing inhibitory current in L4 PNs, suggesting a recruitment of FS neurons by the thalamus. Intracortical stimulation, on the other hand, recruits disynaptic SOM-mediated inhibition (Beierlein et al., 2003). Additionally, stimulation of thalamic projections evokes robust EPSPs in FS INs and RS PNs but only weak excitatory current in SOM neurons in L4 and L5/6 (Beierlein et al., 2003; Cruikshank et al., 2010). Whatever weak thalamic input makes it to SOM neurons is still mediated by depressing rather than facilitating synapses, making thalamic drive to SOM neurons even less impactful (Cruikshank et al., 2010). Within cortex, stimulation of L4 neurons evokes much stronger EPSPs in L2/3 PV cells and PNs than in SOM cells, whereas stimulation of surrounding L2/3 PNs leads to a strong recruitment of L2/3 SOM neurons (Adesnik et al., 2012). These results from visual cortex indicate that SOM cells in L2/3 are modulated more by within-layer input than translaminar input. SOM neurons in L2/3 of auditory cortex tend to have delayed EPSPs during presentation of sound, also suggesting that they are not directly connected to thalamorecepient neurons in L4, but rather pool input from neighboring L2/3 PNs (Li et al., 2015). Weak but facilitating input would take longer to integrate and produce a measurable EPSP, which could explain why excitatory currents in SOM cells are delayed. Although more systematic recordings from different layers and cortical regions are necessary to clarify the nature of the input that drives SOM neurons, it is clear that SOM neurons do not respond with the same dynamics as other neurons.

In visual cortex, different subtypes of interneurons are frequently coactive with other neurons within their class. Thus SOM neurons are more likely to be recruited when other SOM neurons are firing. An interesting pattern emerges when co-inhibition between different inhibitory subclasses and their excitatory input are examined. Subclasses that exhibit strong co-inhibition (such as VIP-SOM) tend to receive non-overlapping excitatory input, whereas those with weak co-inhibition (such VIP-PV) have highly correlated membrane potentials (Karnani et al., 2016b).

## What does the somatostatin neuropeptide do?

Somatostatin is not just a cell-type specific marker, but also an inhibitory 14-amino-acid neuropeptide released by the subset of GABAergic neurons that express the somatostatin gene. Somatostatin activates 5 distinct G-protein coupled receptors (Hoyer et al., 1995). The cellular and synaptic effects of somatostatin are fairly well-understood, but less is known about the network, cognitive, and behavioral effects (for review see Baraban and Tallent, 2004; Liguz-lecznar et al., 2016). Unlike GABA, which is released from conventional synaptic vesicles at axonal boutons, somatostatin is released from dense-core vesicles from both axons, and dendrites (Ludwig and Pittman, 2003). Neuropeptide release requires repetitive high-frequency firing (Kits and Mansvelder, 2000), suggesting that GABA and SOM are likely to be released under different conditions. Indeed, in the hippocampus, GABA and SOM are differentially released during the different oscillatory activities accompanying sleep and movement, suggesting the possibility of distinct functional roles (Katona et al., 2014). The functional interactions of SOM and GABA can be complex. In hippocampus, both SOM and GABA produce postsynaptic hyperpolarization, with SOM augmenting multiple K+ currents and reducing voltage-gated Ca++ currents (Pittman and Siggins, 1981; Moore et al., 1988; Ishibashi and Akaike, 1995; Viana and Hille, 1996; Schweitzer et al., 1998). SOM also acts via presynaptic receptors to inhibit glutamate release by excitatory neurons (Boehm and Betz, 1997; Tallent and Siggins, 1997; Dutar et al., 2002; Sun et al., 2002). Repeated release of SOM also reduces the density of dendritic spines and excitatory synapses, which depends on activation of SOM receptor subtype 4 (Hou and Yu, 2013). All of these actions would be expected to work in concert with GABA release to reduce the firing probability of downstream neurons. However, there is some evidence that SOM also decreases GABA-mediated IPSPs and can lead to depolarization, which would counteract the inhibitory effects of GABA (Dodd and Kelly, 1978; Scharfman and Schwartzkroin, 1989; Greene and Mason, 1996; Leresche et al., 2000). The cellular and synaptic effects of SOM release in cortex have not been studied.

The effects of the somatostatin neuropeptide on network activity and cognition have been studied by intracerebral injections of agonists and antagonists, and also with SOM knockout mice. SOM appears to have an antiepileptic effect, reducing epileptiform activity and seizures in a number of different epilepsy models (Sun et al., 2002; Halabisky et al., 2010). This makes sense because of the inhibitory effects of SOM and the fact that it is released only under conditions of sustained high-frequency firing. Consistent with this, SOM knockout mice show increased severity of kainate-induced and sensory-triggered seizures (Buckmaster et al., 2002; Tomioka et al., 2014). Together these findings suggests that one function of SOM may be to act as a protective neuropeptide system to prevent runaway activity. SOM also appears to play a role in learning and memory. SOM receptor knockout mice show motor (Zeyda et al., 2001) and spatial learning deficits (Guillou et al., 1993; Dutar et al., 2002; Tuboly and Vécsei, 2013), whereas hippocampal or ventricular injections of SOM facilitate spatial learning in a variety of tasks (Vécsei et al., 1984; Vécsei and Widerlov, 1988; Lamirault et al., 2001). Cysteamine, a SOM-depleting agent, leads to deficits in memory retention in rats (Vécsei et al., 1984; Vécsei and Widerlov, 1988; Fitzgerald and Dokla, 1989; Nakagawasai et al., 2003). Cortical expression levels of SOM are reduced in aging in both rats and humans, and this is correlated with learning deficits in rats (Dournaud et al., 1995). SOM levels are also reduced in the brains of Alzheimer's disease patients examined postmortem, specifically in cortical layers 3 and 5 (Davies et al., 1980; Vécsei and Klivenyi, 1995). However, it's worth noting that many of these studies are relatively old and have not been replicated recently, despite broad interest in the topic. Based on the available evidence, it thus appears that SOM has a potential neuroprotective role in preventing epileptic activity, and also appears to be involved in both learning and memory retention.

## Functional and computational roles of SOM cells

### Receptive field properties

In general, the tuning of inhibitory neurons is very similar among different subtypes, and typically a bit more broad compared to excitatory cells. In L2/3, at least, SOM neurons appear to provide inhibition to nearly all PNs in the local neighborhood, and likewise appear to pool input indiscriminately from the local population (Fino and Yuste, 2011). SOM cells are thus very likely to have the same tuning as the net tuning of the local population, such that their effect on the receptive fields of PNs may not be obvious just from measuring their tuning (Li et al., 2015; Karnani et al., 2016a). In visual cortex, there is evidence that SOM cells are more orientation-tuned than PV neurons (Ma et al., 2010). Their frequency tuning in auditory cortex is also sharper than that of PV cells but does not differ from the tuning of excitatory cells (Li et al., 2015). These differences in tuning at the population level are quite small, however, and are much smaller in magnitude than the variability in tuning across individual cells. Together, these results are largely consistent with the idea that SOM cells, like PV cells, pool input from the local population. This “local pooling hypothesis” explains why inhibitory interneurons tend to reflect the general tuning of the area and the neurons they pool from (Kerlin et al., 2010; Fino and Yuste, 2011; Moore and Wehr, 2013). This idea also explains the minor differences in tuning of IN subtypes, since there are some differences in their input and the size of the area they pool from. For example, PV neurons in L4 receive thalamic input as well as excitatory input from surrounding PNs within about 100 μm, and as a result are highly correlated with local network activity (Beierlein et al., 2003; Scholl et al., 2015). PV neurons provide equal inhibition to PNs located both near and far from them (within a range of about 400 μm). VIP cells, in contrast, provide very local columnar inhibition to SOM cells within about 120 μm (Zhang et al., 2014; Karnani et al., 2016a). SOM neurons receive little or no thalamic input, and pool mostly from PNs in the same layer within about 550 μm (Fino and Yuste, 2011; Zhang et al., 2014). The peak of their inhibitory contribution is seen in PNs located approximately 200 μm away, making them ideal inhibitors of competing neural activity (Fino and Yuste, 2011; Zhang et al., 2014). SOM neurons often form disynaptic inhibitory circuits between PNs, thus contributing strongly to lateral inhibition (Silberberg and Markram, 2007; Fino and Yuste, 2011; Adesnik et al., 2012; Zhang et al., 2014). For example, SOM-mediated lateral inhibition contributes to surround suppression in L2/3 visual cortical neurons, conferring tuning for stimulus size (Adesnik et al., 2012). The fact that SOM cells form a disinhibitory network with VIP neurons, which are activated by locomotion, predicts that surround suppression should be modulated by locomotion (Ayaz et al., 2013; Fu et al., 2014). Indeed, locomotion alters spatial integration in V1 in mice, leading to a decrease in surround suppression (Ayaz et al., 2013). This suggests that effects of SOM cells on the receptive fields of neighboring PNs could depend in complex ways on behavioral state or task context, and may not be revealed by studies in anesthetized animals. For example, SOM neurons in visual cortex have been reported to be much less visually responsive under anesthesia (Adesnik et al., 2012). In addition, since SOM cells appear to integrate input from within their own layer, and have unique interlaminar connectivity, it is possible that specific contributions of SOM inhibition to PN receptive field properties might only be seen under conditions in which cortical layers are differentially activated, as is seen for example during habituation in auditory cortex (Kato et al., 2016).

### Divisive and subtractive inhibition

A number of studies have examined the functional role of SOM neurons and their effect on both PNs as well as interneurons using optogenetic activation or suppression of SOM or PV cells (Atallah et al., 2012; Duguid et al., 2012; Lee et al., 2012; Wilson et al., 2012; Cottam et al., 2013; Seybold et al., 2015). A common theme is that SOM cells provide gain control for cortical circuitry, but perhaps not surprisingly, a diversity of results have been reported. Gain control in this context refers to the general enhancement or suppression of PN responses, independent of specific transformations of stimulus selectivity. For example, moderate suppression or activation of PV neurons in layer 2/3 causes a multiplicative scaling up or down of PN responses in visual cortex, without altering their orientation tuning (Atallah et al., 2012; Wilson et al., 2012). Such scaling is referred to as divisive gain control, which is often contrasted with subtractive gain control. In subtractive gain control, all activity (both spontaneous and evoked) is increased or decreased by a constant amount. In contrast to the findings of Atallah and of Wilson, stronger activation of visual PV neurons appears to produce a subtractive instead of divisive effect on PNs, which narrows their orientation tuning instead of leaving it unaffected (Lee et al., 2012). These conflicting results can best be understood in the context of the “iceberg effect,” which refers to the idea that firing rates cannot go below zero (i.e., the water level), which hides the subthreshold tuning curve. Stronger suppression of PN spiking can narrow tuning curves, even with purely divisive inhibition, because of this effect of spike threshold (El-Boustani and Sur, 2014; Lee et al., 2014; Xue et al., 2014). These studies illustrate that inferring the presence of divisive or subtractive inhibition from optogenetic manipulations can be problematic (Kumar et al., 2013; Seybold et al., 2015).

Because PV cells provide fast and powerful proximal inhibition, they appear to be ideally positioned to provide divisive inhibition, whereas the dendritic targeting by Martinotti cells seems better suited to providing subtractive inhibition (Kubota et al., 2015, 2016). Consistent with this, SOM cells provide subtractive inhibition to PNs in olfactory cortex, and moreover provide divisive inhibition to PV cells there (Sturgill and Isaacson, 2015). In visual cortex, conflicting results have been reported for activation of SOM cells. One study found that SOM activation sharpened PN orientation tuning, consistent with subtractive inhibition (Wilson et al., 2012), whereas a similar study reported that SOM activation reduced PN spiking without any effect on tuning, consistent with divisive inhibition (Lee et al., 2012). A key to reconciling these disparate results may lie in the relative timing, size, and durations of sensory and optogenetic stimulation. In particular, inhibition may be more likely to be divisive when it is co-active with strong PN activity, and more likely to be subtractive when PNs and INs are not co-active. Importantly, one of these studies used brief activation (Wilson et al., 2012), whereas the other used prolonged activation (Lee et al., 2012). Brief SOM activation (which would result in less co-activation) produced a subtractive effect, whereas prolonged SOM activation (with more co-activation) produced a divisive effect (Lee and Dan, 2012; El-Boustani and Sur, 2014). Due to their facilitating input and the fact that they pool broadly from PNs, SOM cells are likely to be only weakly activated by brief or small visual stimuli, whereas prolonged and large visual stimuli are likely to strongly activate the SOM network. Indeed, SOM cells were found to provide late, subtractive inhibition to PNs for small visual stimuli, but switched to fast, divisive inhibition for large visual stimuli (El-Boustani and Sur, 2014). This illustrates that whether gain control is divisive or subtractive is dynamic and stimulus-dependent, rather than a fixed property of a given cell type. In addition, SOM activation suppresses PV cells up to twice as powerfully as PNs, which will have its own effects on network activity and PN tuning (Cottam et al., 2013). A diverse mixture of subtractive and divisive effects during activation of SOM or PV neurons is seen in auditory cortex as well, even in simultaneously recorded neurons within the same column (Seybold et al., 2015). This degree of variability in the effects of SOM network perturbations makes sense given the broadly interconnected and recurrent nature of cortical networks. Indeed, modeling of these networks has shown that inhibitory gain control can shift from being divisive to subtractive depending on the spike threshold of PNs and the strength of the optogenetic suppression. This suggests that these forms of gain control may not be determined by the type of interneuron, but rather by intrinsic properties of a target neuron (Seybold et al., 2015). The picture that emerges from these studies is that whether gain control is divisive or subtractive is a flexible and dynamic feature of inhibitory circuits.

Surprisingly, functional properties of SOM neurons in barrel cortex appear strikingly different from those in auditory and visual cortex. Unlike SOM cells in V1, which have unremarkable responses to visual stimuli, SOM cells in L2/3 of S1 are tonically active in the absence of whisker stimulation but become hyperpolarized and cease firing in response to either active or passive whisker stimulation (Gentet et al., 2012). Optogenetic activation of SOM cells during stimulus presentation might therefore produce unnatural results that would be markedly different from what is seen in the non-perturbed circuit. Optogenetic suppression of SOM cells in S1, however, is easier to interpret, and leads to increased burst firing in nearby PNs (Gentet et al., 2012). Tonically active SOM cells likely provide tonic inhibition to cortical neurons, especially to apical dendrites (the distinctive target of Martinotti cells). Tonic inhibition remains poorly understood in cerebral cortex, but has been shown to improve the signal-to-noise ratio in cerebellum, allowing reliable transmission of sensory information (Duguid et al., 2012). Reducing tonic inhibition in cerebellum mainly results in increased spontaneous activity, with little effect on evoked responses, consistent with results seen in barrel cortex (Gentet et al., 2012). In hippocampus, silencing SOM (but not PV) neurons increased the probability of burst spiking in PNs (Royer et al., 2012), similar to the effect seen in barrel cortex. Burst spiking in cortical regions has been hypothesized to carry more information than single spikes (Livingstone et al., 1996; Lisman, 1997) and is associated with improved stimulus detection in the visual system (Guido et al., 1995; Mukherjee and Kaplan, 1995). This suggests a mechanism by which the hyperpolarization of SOM cells by whisker stimulation might enhance sensory information processing.

In cortex, excitation is typically balanced by inhibition that is proportionally scaled depending on the strength of excitatory input (Anderson et al., 2000; Wehr and Zador, 2003; Wilent and Contreras, 2005; Okun and Lampl, 2008). In order for inhibition to scale proportionately with excitation, both sources must receive at least some overlapping sensory input. Since PV neurons, but not SOM cells, receive thalamic input, they appear to be best positioned to provide balanced inhibition via feedforward circuitry, as has been shown in the visual cortex (Xue et al., 2014). SOM cells, on the other hand, seem more likely to provide modulatory inhibitory input that is pooled from the activity of surrounding PN population, hence providing feedback inhibition (Murayama et al., 2009). Both sources likely contribute to balanced inhibition in PNs.

### Gain control by locomotion

Behavioral states can profoundly change how sensory neurons respond to a stimulus (Niell and Stryker, 2010; Kato et al., 2016). A powerful new model for studying these effects has been to study the effects of locomotion on sensory processing, typically by recording stimulus-evoked responses in mice that are free to run on a ball or wheel. The effects of locomotion are strikingly different across sensory systems. In the visual cortex, for example, running enhances neural responses without changing their orientation tuning (Niell and Stryker, 2010). The opposite effects are seen in the auditory cortex, where projections from secondary motor cortex suppress sensory responses during locomotion (Schneider et al., 2014). Similarly, both the neural circuitry and the neuromodulatory systems underlying locomotion effects also appear to differ across sensory regions. In visual cortex, running depolarizes both PNs and inhibitory cells. The resulting increase in both excitation and inhibition in PNs reduces membrane potential variance, and leads to more stimulus-evoked spikes without any increase in spontaneous activity (Polack et al., 2013). Whereas cholinergic input affects membrane potential fluctuations during quiescent periods, the effect of locomotion on membrane potential variance is mostly dependent on noradrenergic input. Interestingly, SOM neurons do not show decreased membrane potential variability during running, suggesting a differential influence of norepinephrine on SOM neurons and PNs (Polack et al., 2013). Different classes of inhibitory neurons show marked differences in how they are modulated by locomotion in the visual cortex. VIP neurons are depolarized throughout the entire running period, while PV cells only respond transiently at the beginning. SOM neurons are typically suppressed during running, and fire mostly at the end of the running period (Fu et al., 2014). These results suggest that the effect of locomotion is mediated by a disinhibitory circuit, in which VIP cells inhibit SOM cells and thereby increase the activity of neighboring PNs. VIP cells are known to be activated by basal forebrain stimulation, via nicotinic acetylcholine receptors (nAChRs). The basal forebrain projects extensively to V1, and nAChR antagonists strongly reduce the locomotion-induced depolarization of VIP cells. These results suggest that cholinergic projections are a key element of the circuitry underlying the locomotion effect in V1, but because nAChR blockade did not completely abolish this effect, there must be additional pathways involved (Fu et al., 2014). Multiple interacting modulatory pathways could explain the apparent contradiction between the results of Polack and of Fu about the relative importance of norepinephrine and acetylcholine for the locomotion effect in V1. Acetylcholine and norepinephrine modulation can interact in complex ways; for example, it is possible that acetylcholine predominantly affects the gain of evoked responses, while norepinephrine produces shifts in baseline activity, as seen in barrel cortex (Constantinople and Bruno, 2011). Interestingly, ACh also affects SOM neurons, but acts through muscarinic receptors (Kawaguchi et al., 1997; Fanselow et al., 2008; Xu et al., 2013), suggesting that the influence of ACh on activity of cortical neurons may be complex and depend on the type of activated receptor and neuronal subtype.

One exciting but still speculative possibility is that this disinhibitory circuit operates in much the same fashion to increase gain during selective attention or similar top-down enhancement. For example, VIP cells have been proposed to mediate attentional enhancement by opening local holes in the blanket of inhibition (Fomby et al., 2014; Zhang et al., 2014; Karnani et al., 2016a). One difference between this idea and the disinhibitory effects of locomotion is that all VIP cells in V1 are activated by running, which is consistent with the observation that the effects of locomotion are distributed broadly across all of visual cortex. This contrasts with the idea of very local disinhibition achieved by activating one or a few VIP cells. It is possible that the same underlying circuitry could operate in these two distinct modes in different functional contexts.

A similar (but not identical) disinhibitory circuit modulates activity in barrel cortex. Unlike V1, somatosensory cortex receives strong M1 input, particularly to VIP cells that express 5HT3aR serotonin receptors (Lee et al., 2013; Fu et al., 2014). While PNs and other types of INs also receive weak input from M1, VIP neurons in all layers are strongly recruited by M1 activation. Whisking reliably activates VIP neurons that in turn suppress SOM activity (Lee et al., 2013). This disinhibitory circuit therefore explains how SOM cells cease their tonic firing and become hyperpolarized during whisking (Gentet et al., 2012). This suggests that the same disinhibitory circuit motif that underlies the locomotion effect in V1 also modulates S1 sensory responses during active whisking, although the source of VIP activation is different in the two systems. Considering that 5HT3aR-expressing VIP cells are strongly depolarized by serotonin and acetylcholine as well, an intriguing possibility is that other behavioral states and contexts could additionally influence sensory responses during whisking (Moreau et al., 2010; Rudy et al., 2011; Lee et al., 2013).

Although auditory neurons also exhibit depolarization and decreased membrane potential variability during running, effects of locomotion on auditory cortex are distinct, since locomotion mostly suppresses sound-evoked responses instead of enhancing them (Schneider et al., 2014). These changes also tend to precede movement, indicating that modulation comes from a motor planning region rather than from muscle feedback. Interestingly, in auditory cortex, M2 projections inhibit PN responses via the PV network, bypassing the VIP → SOM disinhibitory circuit (Schneider et al., 2014). While running desynchronizes auditory cortex and depolarizes PNs, optimal performance on an auditory task is associated with an intermediate state of arousal and hyperpolarized membrane potentials in PNs, in an attentive but quiescent behavioral state (McGinley et al., 2015). Thus the state of arousal falls along a continuum, and different points of this spectrum are likely mediated by different modulatory systems, not all of which involve SOM inhibitory networks. Taken together, these studies demonstrate that each sensory system integrates information about movements through different local and global circuits.

### Salience and behavioral relevance

A number of recent studies have looked at the responses of SOM neurons during more complex forms of contextual stimulus presentation. In auditory cortex, SOM inhibition contributes to stimulus-specific adaptation and habituation. In both of these phenomena, SOM neurons appear to be sensitive to the statistics of stimulus presentation, and suppress the responses to frequently presented tones. However, the time scales and contextual structure of the two paradigms suggests that they engage distinct processes. Stimulus-specific adaptation describes how auditory neurons respond in an “oddball” paradigm, in which a frequent stimulus is interleaved with a rare stimulus. Responses to the frequent stimulus are suppressed, but responses to the rare stimulus are not. Stimulus-specific adaptation is seen across all cortical layers, and in all cell types, including SOM neurons. The phenomenon can be seen in anesthetized animals, and develops within a few presentations of brief tone stimuli (Szymanski et al., 2009; Chen I.-W. et al., 2015; Natan et al., 2015). Suppression of SOM neurons reduces stimulus-specific adaptation in other cortical neurons, increasing PN responses to the frequent tone. Thus SOM cells contribute to stimulus-specific adaptation, even while experiencing stimulus-specific adaptation themselves (Natan et al., 2015). SOM neurons also contribute to a form of habituation to tones that develops over several days. In this paradigm, daily exposure to repeatedly presented long tones (9 s duration) gradually reduces tone-evoked responses in L2/3 PNs. This habituation can be partially relieved if mice are engaged in a sound detection task (Kato et al., 2016). Tone-evoked responses in L2/3 SOM cells are increased as both PN and PV cell responses are decreased, suggested that SOM cells mediate the habituation in other cortical neurons. This increase in SOM responsiveness contrasts with the decreased SOM responsiveness during stimulus-specific adaptation. However, what causes the increase in SOM inhibition is still unclear. One possibility is that frequently-repeated stimuli might increase SOM activation via facilitating synapses. This mechanism could explain short-term stimulus-specific adaptation in cortical neurons, but cannot explain habituation effects that persist across days. This suggests that there could be long-term cellular or synaptic changes that lead to a “memory” of a frequent tone. Another possibility is that SOM cells might receive specific input that is not adapted during habituation paradigms. Interestingly, however, thalamorecipient L4 neurons did not show the habituation seen in L2/3 PNs, which makes them unlikely candidates for enhanced SOM activation (Kato et al., 2016). This is consistent with the fact that L2/3 SOM cells get little or no input from L4 (Li et al., 2015), and also suggests that this form of habituation is not inherited from subcortical auditory structures. Yet another possibility is that enhancement of SOM cell responses during habituation may be generated by top-down input. For example, SOM cells in visual cortex receive weak but measurable input from cingulate cortex (Zhang et al., 2014). Modulation of VIP neurons, as occurs in different behavioral states (Pi et al., 2013; Fu et al., 2014; Karnani et al., 2016a), is also a plausible candidate for the habituation signal.

Inhibition of SOM cells via VIP neurons, as seen during locomotion, also seems to play a central role in modulating SOM activity during task performance and behavioral relevance (Pi et al., 2013; Zhang et al., 2014; Karnani et al., 2016a). Although locomotion changes activity broadly across visual cortex, disinhibitory effects of VIP neurons might also be local to a region of specific tuning (Karnani et al., 2016a). A highly localized disinhibitory network could provide a mechanism for selectively enhancing visual processing in a small part of the visual field, without affecting inhibition in other regions. A similar circuit motif can also enhance the signal-to-noise ratio in cortical neurons during task performance. Indeed, V1 receives strong localized input from the cingulate cortex that enhances VIP activity (Zhang et al., 2014). A top-down control signal from an executive region would be an ideal candidate to selectively modulate visual responses. In auditory cortex, neurons that are tuned to target frequency display enhanced selectivity during performance of a tone-in-noise detection task, while neurons that are tuned to other frequencies suppress their responses (Atiani et al., 2009). All cortical neurons showed a dramatic gain reduction during the task, which, in combination with sharpened receptive fields for target frequency, leads to a dramatic reduction in noise and better task performance (Atiani et al., 2009; Otazu et al., 2009; Sadagopan and Wang, 2010). Although it is unclear whether the VIP → SOM network is responsible for this short-term receptive field plasticity, reinforcement signals are known to activate VIP-positive neurons in auditory cortex, which in turn results in strong suppression of SOM neurons and of a small fraction of PV cells (Pi et al., 2013).

The modulatory signals that underlie differential recruitment of various cell types during task performance are still unclear. In visual cortex, VIP neurons receive modulatory input from cingulate cortex, basal ganglia, and to a weaker extent M1 (Lee et al., 2013; Fu et al., 2014; Zhang et al., 2014). Slice experiments show that ACh increases input resistance in regular and burst spiking VIP and SOM neurons, resulting in increased firing, whereas fast and late spiking cells remain unaffected (Kawaguchi et al., 1997). Moreover, norepinephrine increases spike probability in regular spiking and bursting SOM neurons in rat frontal cortex (Kawaguchi and Shindou, 1998). Additionally, VIP cells belong to a subgroup of neurons that express 5HT3a receptors, thus making them a primary target of serotonergic projections (Rudy et al., 2011). Adding to this complexity, these modulatory inputs also have different effects on cortical activity. ACh and serotonin desynchronize cortical network activity, whereas norepinephrine synchronizes cortical activity (for review see Lee and Dan, 2012). Cortical neurons can also be desynchronized by tonic glutamatergic input from the thalamus (Hirata and Castro-Alamancos, 2010). Interestingly, ACh can synchronize coupled LTS cells, although it is important to remember that LTS cells are only a subset of SOM neurons (Beierlein et al., 2000). Awake behaving mice typically show desynchronized cortical activity, whereas quiet wakefulness, sleep, and anesthesia are associated with synchronized activity featuring up and down states. Evidence from barrel cortex suggest that non-fast-spiking inhibitory cells tend to correlate with the membrane potential of excitatory cells during quiet wakefulness states, but show dramatic increases in depolarization and firing during active whisking, thus suppressing cortical responses (Gentet et al., 2010). By receiving either direct or disynaptic input from other brain regions, SOM-mediated inhibition could play a role in selective attention of sensory processing during task engagement and dictate changes in cortical network activity during specific behavioral states. Norepinephrine and acetylcholine could have opposing effects because the former activates SOM cells, whereas the latter inhibits them through the VIP → SOM circuit. In summary, an intriguing but still speculative possibility is that different neuromodulators might act via SOM networks to promote synchronized or desynchronized network states, in much the same way as they modulate gain during locomotion.

### Learning

SOM neurons also appear to play an important role in memory formation. Classical fear conditioning of a whisker stimulus increases the number of inhibitory synapses in L4 of the corresponding barrel in S1 (Siucinska, 2006; Jasinska et al., 2010). Recently, upregulation of GABA was shown to be accompanied by an increase in the number of SOM-expressing neurons in L4 of barrel cortex after conditioning (Gierdalski et al., 2001; Cybulska-Klosowicz et al., 2013). This presumably results from an increase in SOM expression by SOM cells, bringing them above immunostaining detection threshold, rather than an increase in the number of SOM cells *per se*. Thus SOM cells express more GABA and somatostatin after associative learning, suggesting that increased SOM-mediated inhibition may play a role in circuit plasticity during learning. In cortical slices from naive animals, SOM neurons exhibit lower levels of GABA expression compared to other classes of inhibitory cells (Gonchar and Burkhalter, 1997). Upregulation of GABA after associative learning may allow the SOM inhibitory network to shape cortical responses that represent a newly behaviorally-relevant stimulus after learning. A quite different pattern of changes has been demonstrated in motor cortex, where motor learning induces reorganization of dendritic spines on the apical tufts of L2/3 PNs. This reorganization coincides with a decrease in axonal boutons of SOM cells in layer 1 shortly after the beginning of training (Chen S. X. et al., 2015). Indeed, SOM activation during motor learning destabilizes spines on PN apical tufts, whereas SOM suppression hyperstabilized those spines. These two results—fewer SOM synapses after motor learning, but more synapses after associative learning—appear at first glance to be contradictory. However, the decrease after motor learning was seen for L1 synapses onto L2/3 apical dendrites, whereas the increase after associative learning was seen in L4. The former are almost certainly synapses from Martinotti cells, which target PNs, whereas the relevant L4 SOM cells could be non-Martinotti cells that target PV interneurons in L4. The net effect of these changes could therefore be in the same direction—a disinhibition of PNs. Much remains to be understood about exactly how these SOM inhibitory networks are involved in learning. Yet the differential activation of these networks during learning paradigms illustrates that SOM cells do not merely relay sensory information, but rather modify cortical sensory responses based on an animal's previous experience.

### Challenges in studying interneuron populations

Although new optogenetic tools have been indispensable in understanding the role of specific cell types in intact cortical circuits *in vivo*, they do have important limitations that must be considered when interpreting the results. Variability in illumination intensity and duration, transgenic vs. viral expression, and the details of sensory stimuli or task parameters can interact in complex ways that affect how neurons respond to optogenetic manipulation even in the same cortical region. In the case of Arch-mediated suppression, for instance, depending on the cell type, and region of inactivation, it can be extremely difficult to completely silence neural responses. For example, spontaneous or low-amplitude evoked activity can show 65–80% suppression, whereas strong evoked bursting activity remains unchanged even with high-power Arch activation (Cardin, 2012). Expression of ChR2 can also produce variable responses of a given cell to the same light pulse, ranging from robust spiking responses to less reliable prolonged responses (Cardin, 2012). Misinterpretation of ChR2 manipulation may also come from atypical firing patterns evoked in cells that show late or suppressed responses under control conditions. In S1 SOM cells, for example, which are normally hyperpolarized by sensory stimulation, optogenetic activation would produce highly unnatural activity (Gentet et al., 2012). Recent advances in genetic calcium imaging techniques (such as GCaMP) have provided a new approach to the study of inhibitory neuronal populations such as SOM networks ((Jackson et al., 2016; Karnani et al., 2016a),b). Two caveats to this approach are that SOM cells are known to have high basal firing rates, and TdTomato labeling in high concentrations can contaminate the fluorescence signal. Both of these caveats could lead this technique to underestimate neuronal firing rates, and thus care should be taken when inferring neural activity from fluorescence changes. Lastly, high levels of interconnectivity in cortical circuits make it challenging to study the effects of only a single interneuron subtype. In particular, it is virtually impossible to perturb the activity of the SOM network without changing firing patterns in other inhibitory neurons, which may lead to conflicting and misleading results (Gibson et al., 1999; Cottam et al., 2013). Nevertheless, the past few years have seen tremendous advances in knowledge about interneuron function in general, and SOM cell function in particular. It is important to remember that SOM cells are not a single population, but rather consist of several distinct classes of inhibitory cells; new methods for targeting these specific subpopulations will only accelerate discoveries in the near future. It is also exciting that much of the progress in recent years has come not from studying SOM cells and sensory cortex in isolation, but rather from taking account of behavioral state, task context, learning, and sensorimotor integration—in other words, how SOM cells participate in large-scale interactions between sensory cortex and the rest of the brain.

## Author contributions

IY and MW wrote the paper.

## Funding

This work was funded by NIH R01 DC011379.

### Conflict of interest statement

The authors declare that the research was conducted in the absence of any commercial or financial relationships that could be construed as a potential conflict of interest.
